# Annexin A2 contributes to cisplatin resistance by activation of JNK-p53 pathway in non-small cell lung cancer cells

**DOI:** 10.1186/s13046-017-0594-1

**Published:** 2017-09-08

**Authors:** Xiaomin Feng, Hao Liu, Zhijie Zhang, Yixue Gu, Huisi Qiu, Zhimin He

**Affiliations:** Cancer Hospital and Cancer Research Institute, Guangzhou Medical University, Guangzhou, No.78 hengzhigang Road, Guangzhou, 510095 People’s Republic of China

**Keywords:** Annexin A2, Cisplatin resistance, Apoptosis, p53, JNK, C-Jun, Non-small cell lung cancer

## Abstract

**Background:**

Development of resistance to therapy continues to be a serious clinical problem in lung cancer management. We previously identified that Annexin A2 is significantly up-regulated in cisplatin-resistant non-small cell lung cancer (NSCLC) A549/DDP cells. However, the exact function and molecular mechanism of Annexin A2 in cisplatin resistance of NSCLCs has not been determined.

**Methods:**

Western blot and qRT-PCR were performed to analyze the protein and mRNA level of indicated molecules, respectively. Immunohistochemistry was performed to analyze the expression of Annexin A2 in NSCLC tissue samples. MTS assay, Colony formation assays, AnnexinV/PI apoptosis assay, Luciferase Reporter Assay, Chromatin-immunoprecipitation, and nude mice xenograft assay were used to visualize the function of Annexin A2 on cisplatin resistance.

**Results:**

Our results demonstrated that knockdown of Annexin A2 increased cisplatin sensitivity of cisplatin-resistant A549/DDP cells both in vitro and in vivo, whereas overexpression of Annexin A2 increased cisplatin resistance of A549, H460 and H1650 cells. Moreover, we found that Annexin A2 enhanced cisplatin resistance via inhibition of cisplatin-induced cell apoptosis. Our studies showed that Annexin A2 suppressed the expression of p53 through activation of JNK/c-Jun signaling, which in turn resulted in a decrease in the expression of p53-regulated apoptotic genes p21, GADD45 and BAX, as well as p53-dependent cell apoptosis. Furthermore, we found that in NSCLC cases that Annexin A2 is highly expressed; it is positively correlated with a poor prognosis, as well as correlated with short disease-free survival for patients who received chemotherapy after surgery.

**Conclusions:**

These data suggested that Annexin A2 induces cisplatin resistance of NSCLCs via regulation of JNK/c-Jun/p53 signaling, and provided an evidence that blockade of Annexin A2 could serve as a novel therapeutic approach for overcoming drug resistance in NSCLCs.

**Electronic supplementary material:**

The online version of this article (10.1186/s13046-017-0594-1) contains supplementary material, which is available to authorized users.

## Background

Lung cancer is a leading cause of cancer-related deaths worldwide, and non-small-cell lung cancer (NSCLC) accounts for approximately 80% of all cases of lung cancer [1]. The majority of patients with advanced lung cancer are treated with chemotherapy [2]. Platinum-based drugs, particularly cisplatin (DDP), are used in the treatment of many cancers, including NSCLC [3]. Initially, cisplatin treatment demonstrates favorable outcomes, but often chemo-resistance develops later on and results in failure of this therapy [4]. Thus, a better understanding of the molecular mechanisms underlying cisplatin resistance is mandatory to achieve advancement in NSCLC therapy.

Annexin A2 is a calcium-dependent, phospholipid-binding cell surface protein which has diverse cellular functions, including membrane-cytoskeleton organization, vesicular trafficking, and regulation of ion channel activity [5]. Accumulating evidence suggested a correlation between the deregulation of Annexin A2 expression and tumor progression in many cancers [6]. High level of Annexin A2 was detected in various malignant tumors, including brain tumors [7], colorectal cancer [8], gastric cancer [9], pancreatic cancer [10], breast cancer [11], hepatocellular cancer [12], and lung cancer [13], and this abnormal expression of Annexin A2 was positively correlated with the differentiation status, histological type, lymph node metastasis and distant metastasis, as well as poor prognosis [14, 15]. Annexin A2 has been shown to play an important role in cancer cell adhesion, proliferation, invasion, and metastasis, thus playing a crucial role in tumor development. Moreover, recently studies showed that Annexin A2 appears to be involved in the drug resistance phenotype of cancer cells. Quantitative proteomics results indicated that Annexin A2 was up-regulated in Adriamycin-resistant MCF-7 cells compared to drug-sensitive MCF-7 cells [16], and up-regulation of Annexin A2 played a critical role in enhanced invasiveness of MCF-7/ADR cells [17]. Annexin A2 overexpression was also significantly associated with rapid recurrence after gemcitabine-adjuvant chemotherapy in patients with resected pancreatic cancer [18, 19]. In our previous studies, we found that Annexin A2 is up-regulated in the cisplatin-resistant non-small cell lung cancer cell line A549/DDP [20]. However, the role of Annexin A2 in cisplatin resistance has not been determined.

In this study, we investigated the role of Annexin A2 in cisplatin resistance of NSCLC cells by analyzing its function both in vitro and in vivo. Our research demonstrated for the first time that Annexin A2 contributes to cisplatin resistance by activation of JNK-p53 pathway in lung cancer cells, and suggested promise as a marker for patients likely to benefit from cisplatin-based chemotherapy.

## Materials and methods

### Cell lines and cell culture

A549 and cisplatin-resistant A549/DDP cells were purchased from the Cancer Institute & Hospital, Chinese Academy of Medical Sciences (Beijing, China). H460 and H1650 cells were purchased from the American Type Culture Collection (Manassas, VA, USA). The cell lines were tested for authenticity by short tandem repeats (STR) genotyping. Cells were maintained in Dulbecco’s Modified Eagle Medium (DMEM) (Gibco, Grand Island, New York State, USA) supplemented with 10% fetal bovine serum (Gibco), 100 U/mL penicillin and 100 mg/mL streptomycin. Cell lines were incubated at 37 °C in a humidified atmosphere of 5% CO_2_. A549/DDP cells were cultured in medium supplemented with cisplatin at a final concentration of 2 μM, to maintain drug resistance.

### Reagents and antibodies

Cisplatin was purchased from Sigma Aldrich (Sigma-Aldrich, St. Louis, USA). Specific inhibitors of AKT (LY294002), JNK (SP600125), and p38 (SB203580) were purchased from Selleck Chemicals (Houston, TX, USA) and dissolved in DMSO. The list of antibodies used in this study is summarized in Additional file 1: Table S1.

### RNA interference, lentivectors, and plasmid transfection

Negative control siRNA and siRNAs against Annexin A2 (human Annexin A2 siRNA) were produced by RiboBio Co. Ltd. (Guangzhou, China). And targeting sequences were 5′-CAAGCCCCTGTATTTTGCTGAT-3′ (Annexin A2 siRNA-1), 5′-CGGGAT GCTTTGAACATTGAA-3′ (Annexin A2 siRNA-2), 5′- GCAGGAAATTAACAGAG TCTA-3′ (Annexin A2 siRNA-3). The small-interfering RNA (siRNA) targeting p53 (SignalSilence® p53 siRNA I #6231) were from Cell Signaling Technology (Beverly, MA, USA). A549/DDP cells were plated into 6-well plate at a density of 1.5 × 10^5^ cells per well. After 24 h of incubation, negative control siRNA, siRNAs targeting human Annexin A2 or p53 were transiently transfected to A549/DDP cells with Lipofectamine 2000 Transfection Reagent (Invitrogen, California, USA) and incubated for 48 h. Annexin A2 shRNA-lentivirus vectors and a non-targeting control shRNA were obtained from GeneCopeia (Guang zhou, China). HEK293FT cells were co-transfected with lentivirus and packaging vectors using Lipofectamine 2000. After 48 h, media containing lentiviruses were harvested and filtered through 0.45 μm syringe filters. Lentiviruses were transduced in 50% confluent A549/DDP cells. Annexin A2 stable knockdown A549/DDP cells were selected by puromycin (1 μg/mL). For Annexin A2 rescue experiments, 1 μg of empty vector (pCMV6) or pCMV6-Annexin A2 (Origene, Rockville, MD, USA) were transfected in A549 cells using Fugene (Roche, Indianapolis, IN, USA), following the manufacturer’s instructions.

### Cell viability assay

Cell viability was measured using the 3-(4, 5-Dimethylthiazol- 2-yl)-2, 5- Diphenyltetrazolium Bromide (MTT) assay. Briefly, cells (1 × 10^4^) were seeded in 96-well plates. Cells were allowed to adhere for 24 h and were subsequently incubated with the indicated drug concentrations for 48 h. 100 μL of 0.25 mg/mL MTT in medium culture was added to each well. The plate was incubated for 4 h at 37 °C. Then, culture medium was removed, and DMSO (100 μL) was added into each well to dissolve the dark blue crystal. The amount of MTT formazan product was analyzed by microplate reader (Spectra MAX 340, Molecular Devices Co., Sunnyvale, CA, USA) at a wavelength of 570 nm. Each individual experiment was repeated at least three times.

### Colony formation assays

For colony-formation assay, about 500 cells were seeded per well in six-well-plates 48 h before the addition of indicated chemicals. After 14 days, the cells were fixed in methanol and stained with 0.2% crystal violet. Number of colonies was counted using Quantity One software (Bio-Rad, Hercules, CA, USA).

### Cell apoptosis assay

Cell apoptosis was determined by AnnexinV/PI (KeyGEN, Nanjing, China), followed by flow cytometer analysis (Beckman Coulter, California, USA) according to manufacturer’s instructions.

Caspase activity was measured by Caspase-Glo 3/7 Assay kit (Promega, Madison, WI, USA) according to the manufacturer’s protocol.

### Western blot

Cells were harvested and proteins were extracted using RIPA buffer (50 mM Tris-Cl, pH 8.0, 150 mM NaCl, 5 mM EDTA, 0.1% SDS, 1% NP-40) supplemented with protease inhibitor cocktail. Cell lysates were centrifuged at 12000 rpm for 30 min at 4 °C, supernatants were saved, and protein concentrations were determined by by BCA protein assay (Thermo Scientific, Rockford, Illinois, USA). Protein extracts (40 μg) was resuspended and electrophoresed on 10–12% sodium dodecyl sulfate polyacrylamide gel and then blotted onto polyvinylidene fluoride membranes (Millipore A) at 200 mA for 1.5 h. Following blocking with 5% nonfat milk in TBST (TBS-1% Tween 20) for 1 h, membranes were immunoblotted with primary antibodies overnight at 4 °C and further incubated with secondary horseradish peroxidase-conjugated anti-rabbit. Finally, protein bands were detected by developing the blots with the enhanced chemiluminescence western blot detection kit (Engreen Biosystem, China).

### RNA extraction and quantitative real-time PCR

Total RNA isolated from cells was used E.Z.N.A.® HP Total RNA Kit (Omega Bio-tek, Doraville, GA, USA). The reverse transcription was performed with the PrimeScript® RT reagent Kit (TakaRa, Shiga, Japan). After mixing the resulting Complementary DNA template with primers, respectively and TaKaRa SYBR® Premix Ex Taq™, Quantitative Real-Time PCR reaction was performed on a ABI 7500 Fast Real-Time PCR System (Applied Biosystems, Foster City, CA). Gene-specific primer pairs used in this study are: p53, 5′-GCGCACAGAGGAAGAGAATCTCCG-3′ (sense) and 5′-TTTGGCTGGGGAGAGGAGCTG-3′ (antisense); GADD45A, 5′-GGATGCCCTGGAGGAAGTGCT-3′ (sense) and 5′-GGCAGGATCCTTCCATTGAGATGAATGTG-3′ (antisense); p21 5′-TGTACCCTTGTGCCTCGCTC-3′ (sense) and 5′-TGGAGAAGATCAGCCGGCGT-3′ (antisense); BAX: 5′-TTTGCTTCAGGGTTTCATCC-3′ (sense) and 5′-CAGTTGAAGTTGCCGTCAGA-3′ (antisense); Bcl2: 5′-GGATGCCTTTGTGGAACTGT-3′ (sense) and 5′-AGCCTGCAGCTTTGTTTCAT-3′ (antisense); MDM2: 5′-AGCGCAAAACGACACTTACA-3′ (sense) and 5′-ACACAATGTGCTGCTGCTTC-3′ (antisense); β-actin: 5′-ACACTGTGCCCATCTACGAGG-3′ (sense); β-actin 5′-AGGGGCCGGACTCGTCATACT-3′ (antisense). β-actin was used as the reference gene. The relative levels of gene expression were represented as ΔCt-Ct gene-Ct reference, and the fold change of gene expression was calculated by the 2^−ΔΔCt^ Method. Experiments were repeated in triplicate.

### Chromatin-immunoprecipitation

The ChIP assay was carried out using Millipore EZ-Magna ChIP kit (Millipuro) following the manufacturer’s instruction. Briefly, 5 × 10^6^ cells were fixed with 1% formaldehyde and quenched in 0.125 M glycine. Cells were sonicated by Bioruptor Sonication System UCD-300. DNA was immunoprecipitated by either control IgG or c-Jun antibody. Precipitated DNA samples and inputs were amplified by PCR. The primers used for the amplification of c-Jun binding site in p53 promoter are: 5′-GCTGAGAGCAAACGCAAAAG-3′ (sense) and 5′-GAAATGGAGTTGGGGAGGAG-3′ (antisense).

### Luciferase assays

The p53 promoter (−540 to +160 regions) was inserted into the pGL3-basic vector (Promega, Madison, WI, USA) at KpnI/XbaI sites to construct pGL3-p53 luciferase reporter plasmid (wild-pGL3-p53). The PF-1 site of the p53 promoter was mutated (from 5′-TGACTCT-3′ to 5′-TGAATTC-3′) using the QuikChange Site-directed mutagenesis kit (Stratagene, La Jolla, CA), and then the PF-1 site-mutated p53 promoter was cloned into the pGL3-basic vector (mut-pGL3-p53). All constructs were verified by sequencing. For the luciferase reporter assay, HEK293T cells and A549 cells were co-transfected with pGL3-p53-Luc, or mut-pGL3-p53 and pCMV-Annexin A2 using Lipofectamine 2000. The Renilla luciferase was as internal control. 48 h after transfection, cells were harvested and assayed by the Dual-Luciferase Reporter Assay System (Promega, Madison, WI, USA). The relative firefly luciferase activity was calculated by normalizing transfection efficiency according to the Renilla luciferase activity.

### Animal study

24 female BABL/c nude mice (4–5 weeks old) from the Guangdong Province Laboratory Animal Center (Guangzhou, China) were used. A549/DDP/Control shRNA cells and A549/DDP/Annexin A2 shRNA cells (1 × 10^6^) suspended in 100 μl cold PBS were subcutaneously injected to the right shoulder of the mice, all of which developed tumors with a size of ~30 mm^3^ within 7 days. Both A549/DDP/Control shRNA cells-bearing mice and A549/DDP/Annexin A2 shRNA cells-bearing mice were randomly allocated to two groups and treated with either PBS or Cisplatin (3 mg/kg body weight per day). The tumor size was measured in two dimensions with a caliper. Tumor volume was calculated based on the following formula: volume = (greatest diameter) × (smallest diameter)^2^/2. After treatment for 4 weeks, the tumors were extracted from sacrificed mice. Our animal study was approved by the Institutional Animal Care and Use Committee of Guangzhou medical University.

### Immunohistochemistry

152 tumor tissues and 36 adjacent normal tissues were obtained from NSCLC patients who underwent complete resection in the Affiliated Tumor Hospital of Guangzhou Medical University between 2008 and 2013. In particular, adjacent normal tissues were taken 5–10 cm away from the tumor tissues. Follow-up information was obtained from review of the patients’ medical record. This study was approved by the Ethics Committee of Guangzhou Medical University.

Immunostaining was performed using the avidin-biotin-peroxidase complex method (UltrasensitiveTM, MaiXin, Fuzhou, China). The sections were deparaffinized in xylene, rehydrated with graded alcohol, and then boiled in 0.01 M citrate buffer (pH 6.0) for 2 min with an autoclave. Hydrogen peroxide (0.3%) was applied to block endogenous peroxide activity, and the sections were incubated with normal goat serum to reduce nonspecific binding. Tissue sections were incubated with Annexin A2 rabbit polyclonal antibody (1:100 dilutions). Staining for antibody was performed at room temperature for 2 h. Biotinylated goat antimouse serum IgG was used as a secondary antibody. After washing, the sections were incubated with streptavidin-biotin conjugated with horseradish peroxidase, and the peroxidase reaction was developed with 3,3′-diaminobenzidine tetrahydrochloride.

The intensity of Annexin A2 staining was scored as 0 (no signal), 1 (weak), 2 (moderate), and 3 (marked). Percentage scores were assigned as 1, 1–25%; 2, 26–50%; 3, 51–75%; and 4, 76–100%. The scores of each tumor sample were multiplied to give a final score of 0–12, and the tumors were finally determined as negative (−), score 0; lower expression (+), score ≤ 4; moderate expression (++), score 5–8; and high expression (+++), score ≥ 9. An optimal cutoff value was identified: a staining index of five or greater was used to define tumors of high expression, and four or lower for low expression.

### Bioinformatics analysis

The prognostic value of the Annexin A2 was analyzed by a Web-based Kaplan–Meier plotter (http://www.kmplot.com/lung), which is a meta-analysis tool of gene expression and survival data of 2437 lung cancer patients (2015 version) using multiple microarray data [21, 22].

### Statistical analysis

All experiments were performed in triplicate at least. The results of this study are presented as mean ± SD and analyzed by Student’s t test with statistical analysis of data using GraphPad Prism 5 (GraphPad Software Inc., San Diego, CA).

## Results

### Annexin A2 enhances cisplatin resistance of NSCLCs

**Fig. 1:**
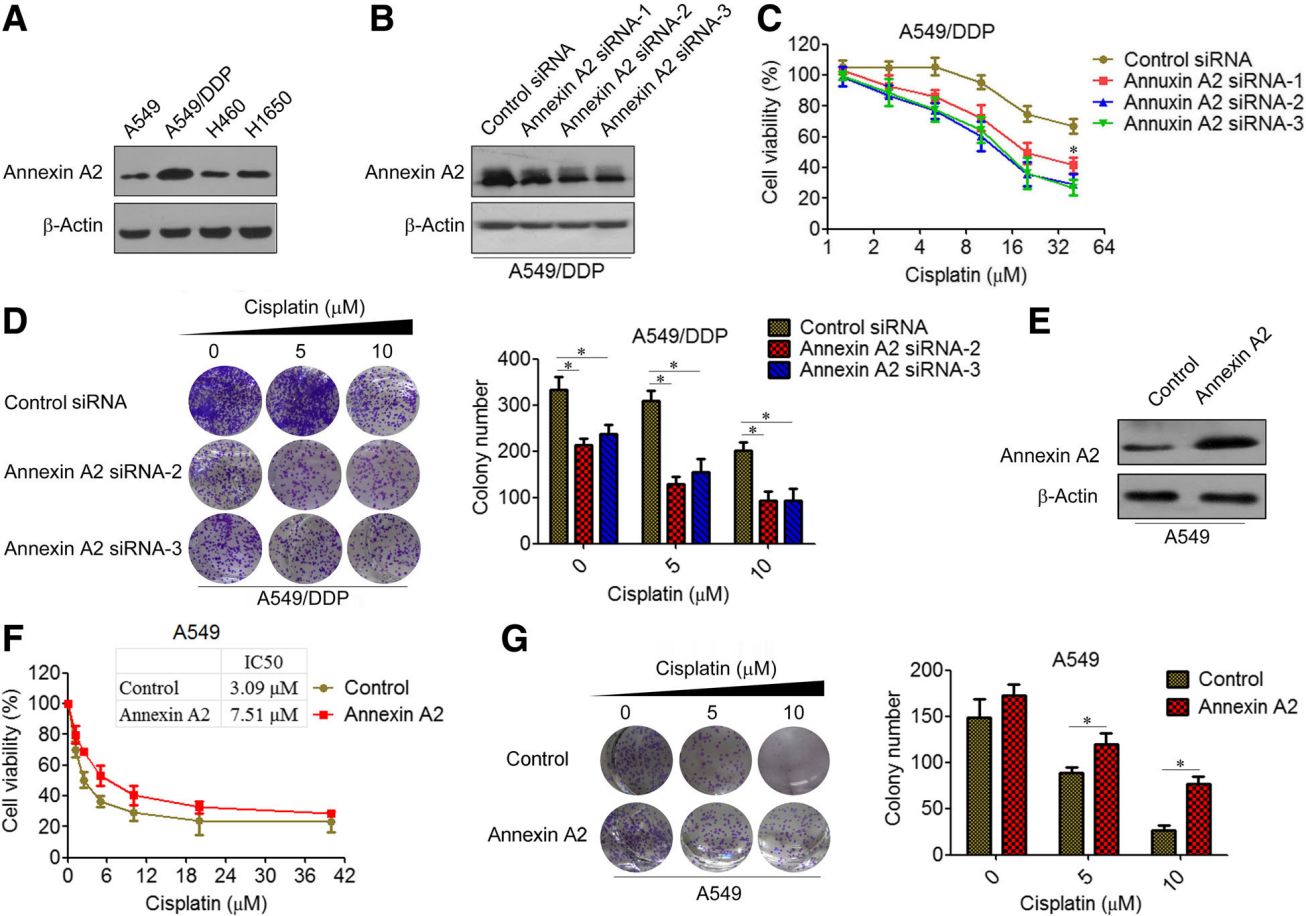
Annexin A2 enhances cisplatin resistance of lung cancer cells. **a** Annexin A2 expression of A549/DDP, A549, H460 and H1650 cells was analyzed by Western blot. β-Actin was employed as an inner control. **b** A549/DDP cells were transfected with Annexin A2 siRNA, Annexin A2 expression was analyzed by Western blot. **c** A549/DDP cells transfected with Annexin A2 siRNA were treated with cisplatin at the indicated concentration for 48 h, cell viability was measured by MTT assay. **d** A549/DDP cells transfected with Annexin A2 siRNA were treated with cisplatin at the indicated concentration for 14 days, (Left) Colonies were fixed with acetic acid-methanol (1:4) and stained with crystal violet. (Right) The number of colonies was from three independent experiments. **e** A549 cells were transfected with pCMV6-Annexin A2, Annexin A2 expression was analyzed by Western blot. **f** A549 cells transfected with pCMV6-Annexin A2 were treated with cisplatin at the indicated concentration for 48 h, cell viability was measured by MTT assay. Table indicates the IC50 values for each condition. **g** A549 cells transfected with pCMV6-Annexin A2 were treated with cisplatin at the indicated concentration for 14 days, (Left) Colonies were fixed with acetic acid-methanol (1:4) and stained with crystal violet. (Right) The number of colonies was from three independent experiments. *P < 0.05

Previously, we compared the protein expression profiles between cisplatin-resistant A549/DDP cells and A549 cells by using a 2-DE proteomic approach, and successfully identified 46 differentially expressed expression by MALDI-TOF-MS/MS [20]. We found that Annexin A2 was significantly up-regulated in A549/DDP cells compared with control A549 cells (Additional file 2: Figure S1). To validate the proteomics results, we performed western blot analyses to determine the expression levels of Annexin A2 in a panel of NSCLC cells, including A549, H460, H1650, and cisplatin-resistant A549/DDP cells. MTS assay showed that A549/DPP cells exhibits more resistant to cisplatin as compared to the A549, H460 and H1650 cells as determined by their IC50 (Additional file 2: Figure S2). Indeed, consistent with the results of 2-DE, the protein expression of Annexin A2 was significantly elevated in A549/DDP cells compared to A549, H460 and H1650 cells (Fig. 1a). Thus, we evaluated the role of Annexin A2 in the maintenance of cisplatin resistance in A549/DDP cells. We found that knockdown of Annexin A2 by using specific siRNAs against Annexin A2 significantly increased cisplatin sensitivity of A549/DDP cells compared with control siRNA (Fig. 1b, c). In clonogenic assays, we also found that silencing of Annexin A2 in combination with cisplatin caused a marked inhibition of proliferation in A549/DDP cells (Fig. 1d). As a complement to RNAi experiments, we assessed the impact of Annexin A2 overexpression on cisplatin sensitivity of A549 cells (Fig. 1e). MTS assay showed that overexpression of Annexin A2 significantly increased cisplatin resistance of A549 cells. The IC50 value of cisplatin for A549/Annexin A2 cells and A549/Control cells were 7.51 μM and 3.09 μM, respectively (Fig. 1f). Similarly, we found that overexpression of Annexin A2 significantly increased cisplatin resistance of H460 and H1650 cells, and the IC50 value for H460/Annexin A2 and H1650/Annexin A2 cells was higher than in control cells (Additional file 2: Figure S3A). Moreover, colony formation assays showed that overexpression of Annexin A2 increased cisplatin resistance of A549, H1650 and H460 cells (Fig. 1g, Additional file 2: Figure S3B). Collective, these results suggested that up-regulation of Annexin A2 is involved in cisplatin resistance of NSCLCs.

### Knockdown of Annexin A2 increased cisplatin sensitivity in vivo

**Fig. 2:**
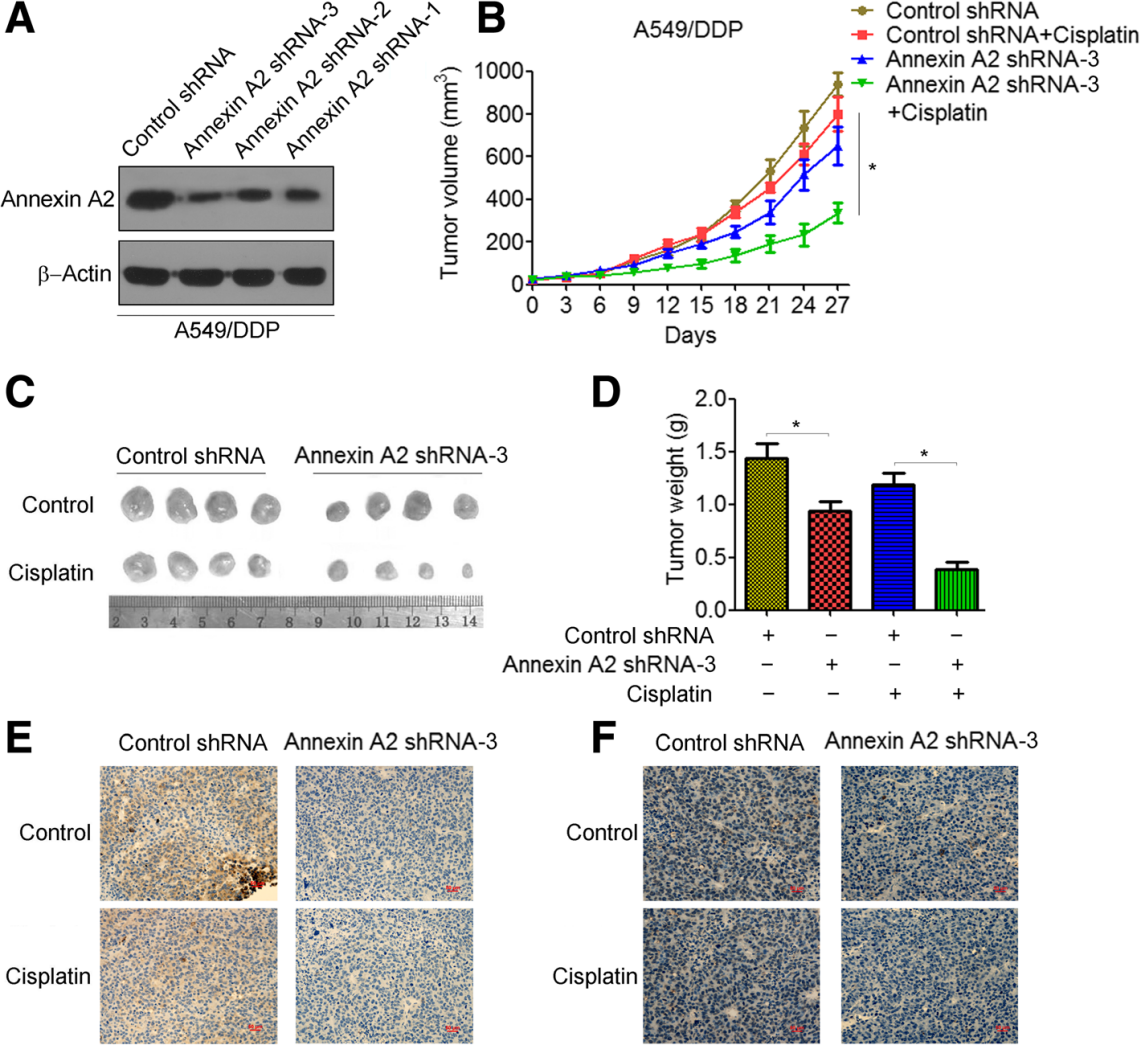
Knockdown of Annexin A2 increased cisplatin sensitivity in vivo. **a** A549/DDP cells were transfected with Control shRNA or Annexin A2 shRNA, Annexin A2 expression was analyzed by Western blot. **b**-**d** A549/DDP cells transfected with Control shRNA or Annexin A2 shRNA were injected to the right shoulder of nude mice. Tumor-bearing mice were treated with either PBS or Cisplatin (3 mg/kg body weight per day) for 4 weeks, tumor sizes were measured at every 3 days (**b**). At the end of treatment, tumor were excised (**c**) and tumor weight were measured (**d**). **e**-**f** Tumor tissues were resected, fixed, sectioned, and placed on slides. Tumor specimens were subjected to immunohistochemical staining with antibodies specific to Annexin A2 (**e**) and Ki67 (**f**). *p < 0.05

Using Annexin A2 shRNA vectors to establish Annexin A2 stably knockdown A549/DDP cells (Fig. 2a), we further investigated the effects of Annexin A2 knockdown on cisplatin resistance in a xenograft tumor model. We found that knockdown of Annexin A2 moderately inhibited tumor growth. More important, knockdown of Annexin A2 significantly increased the drug-sensitization of cisplatin in vivo, in that the tumor volume in A549/DDP/Annexin A2 shRNA-bearing mice treatment with cisplatin was significantly less than that in A549/DDP/Control shRNA-bearing mice (337.4 ± 108.5 mm^3^ vs. 799.6 ± 104.8 mm^3^) (Fig. 2b). The excised tumors from the A549/DDP/Control shRNA-bearing mice treatment with cisplatin weighted between 0.86 g and 1.39 g, whereas these from the A549/DDP/Annexin A2 shRNA-bearing mice treatment with cisplatin averaged ~0.39 g (Fig. 2c, d). Moreover, immunohistochemical staining showed that the expressions of Annexin A2 (Fig. 2e), as well as Ki67 (Fig. 2f), were decreased in the 549/DDP/Annexin A2 shRNA tumors compared to A549/DDP/Control shRNA tumors. These studies indicated that inhibition of Annexin A2 increased cisplatin sensitivity of A549/DDP cells in vivo.

### Annexin A2 confers cisplatin resistance is associated with decreased apoptosis

**Fig. 3:**
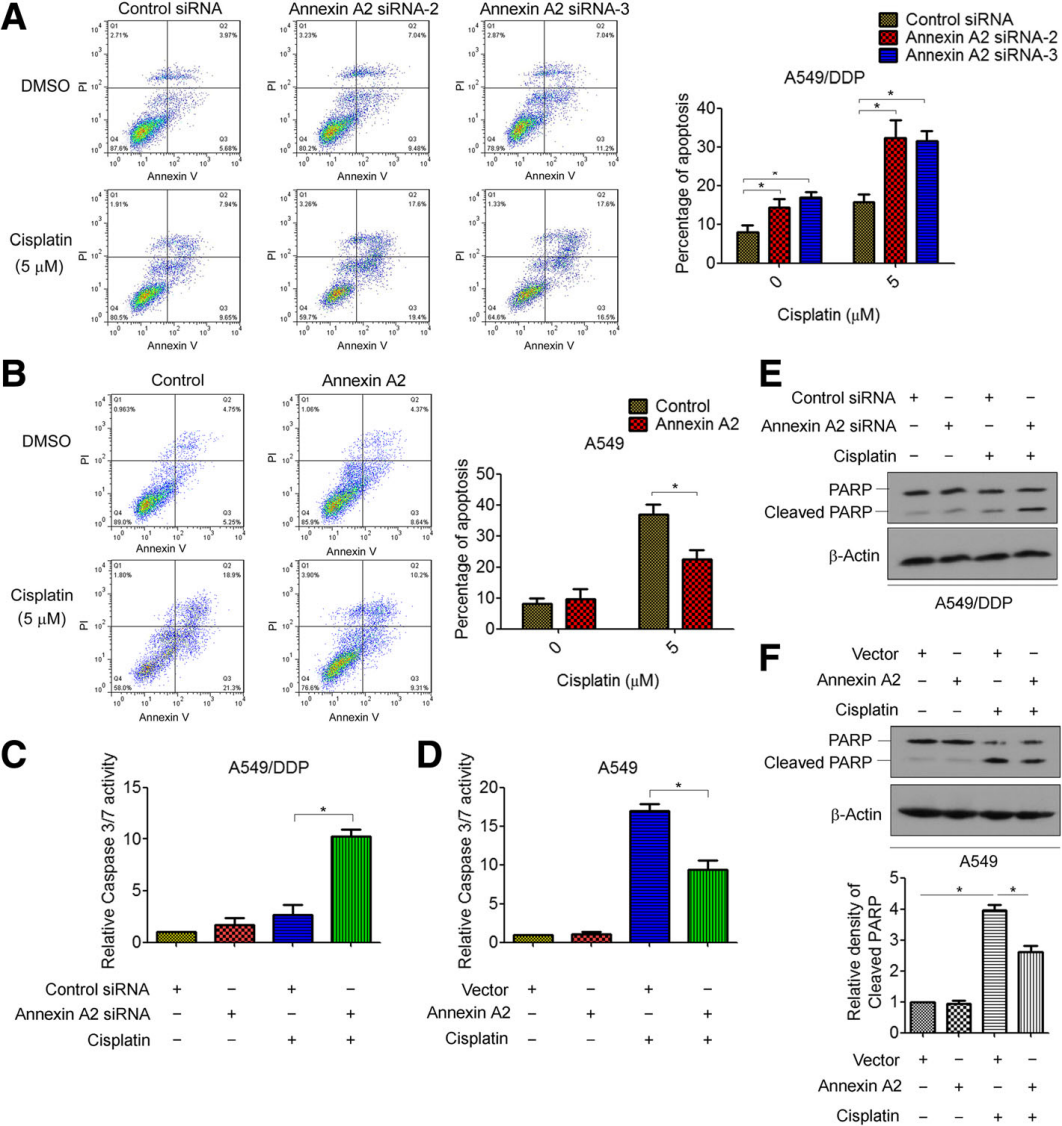
Annexin A2 confers cisplatin resistance is associated with decreased apoptosis. **a** A549/DDP cells were transfected with Annexin A2 siRNA, and then treated with 5 μM cisplatin for 48 h, cells were stained with Annexin V-FITC and propidium iodide, and the cell apoptosis were analyzed by flow cytometry. **b** A549 cells were transfected with pCMV6-Annexin A2, and then treated with 5 μM cisplatin for 48 h, cells were stained with Annexin V-FITC and propidium iodide, the cell apoptosis were analyzed by flow cytometry. **c** A549/DDP cells were transfected with Annexin A2 siRNA, and then treated with 5 μM cisplatin for 24 h, Caspase 3/7 activity were measured. **d** A549 cells were transfected with pCMV6-Annexin A2, and then treated with 5 μM cisplatin for 24 h, Caspase 3/7 activity were measured. **e** A549/DDP cells were transfected with Annexin A2 siRNA, and then treated with 5 μM cisplatin for 48 h, the expression of PARP and cleaved PARP were measured by Western blot. **f** A549 cells were transfected with pCMV6-Annexin A2, and then treated with 5 μM cisplatin for 48 h, (upper) the expression of PARP and cleaved PARP were measured by Western blot, (lower) the relative density of Cleaved PARP. *P < 0.05

Drug resistance often prevents tumor cells from undergoing sufficient levels of programmed cell death or apoptosis, resulting in cancer cell survival and treatment failure [23]. We then investigated whether Annexin A2 confers cisplatin resistance is associated with decreased apoptosis. As shown in Fig. 3a, knockdown of Annexin A2 increased cisplatin-induced cell apoptosis compared with controls in A549/DDP cells. In contrast, overexpression of Annexin A2 significantly attenuated cisplatin-induced apoptosis in A549 cells (Fig. 3b). To further confirm that Annexin A2 restores cisplatin-induced apoptosis, we characterized effects of Annexin A2 on activity of key apoptosis executioner caspase 3/7. A549/DDP cells transfected with control siRNA showed a slight increase in caspase 3/7 activity after treatment of cisplatin. However, cells transfected with Annexin A2 siRNA showed a significant increase in caspase 3/7 activity when incubated with cisplatin at the same concentrations (Fig. 3c). Moreover, overexpression of Annexin A2 significantly decreased cisplatin-induced caspase 3/7 activity in A549 cells (Fig. 3d), as well as in H460 and H1650 cells (Additional file 2: Figure S4A). Furthermore, western blot analysis showed that knockdown of Annexin A2 significantly increased cisplatin-induced cleaved PARP in A549/DDP cells (Fig. 3e), whereas overexpression of Annexin A2 reduced cisplatin-induced cleaved PARP in A549, H460 and H1650 cells (Fig. 3f, Additional file 2: Figure S4B). Therefore, our data suggested that Annexin A2 enhanced cisplatin resistance of NSCLC cells by a mechanism of inhibiting cell apoptosis.

### Inhibition of p53 is critical for Annexin A2-mediated cisplatin resistance

**Fig. 4:**
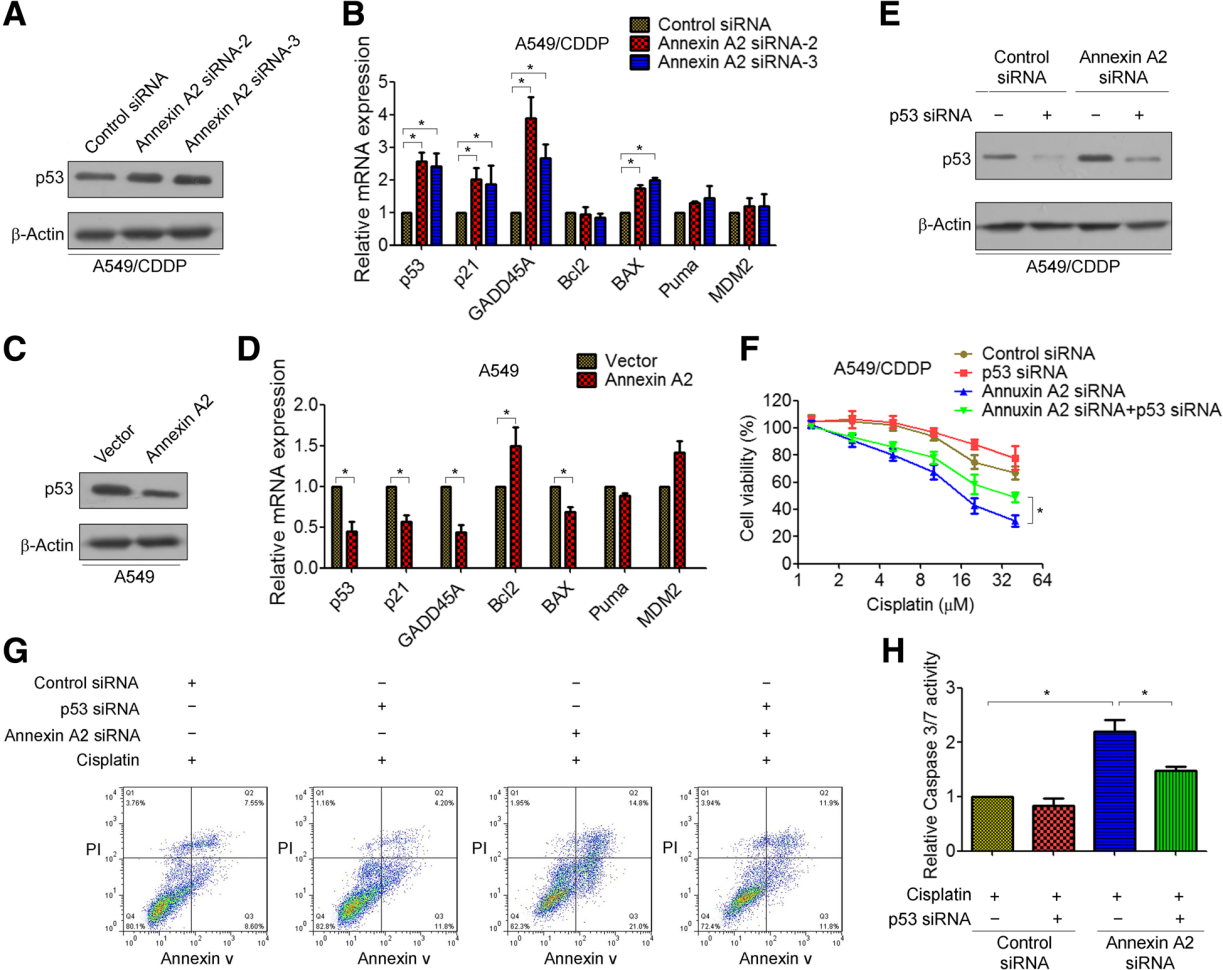
Inhibition of p53 is critical for Annexin A2-mediated cisplatin resistance. **a**-**b** A549/DDP cells were transfected with Annexin A2 siRNA, **a** p53 protein expression was analyzed by Western blot; **b** the expression of p53 and p53-regulated apoptotic genes p21, GADD45, Bcl2, BAX, Puma, and MDM2 were measured by Real time RT-PCR. **c-d** A549 cells were transfected with pCMV6-Annexin A2, **c** p53 protein expression was analyzed by Western blot; **d** p53 and p53-regulated apoptotic genes p21, GADD45, Bcl2, BAX, Puma, and MDM2 were measured by Real time RT-PCR. **e** A549/DDP cells were co-transfected with Annexin A2 siRNA and p53 siRNA, p53 protein expression was analyzed by Western blot. **f** A549/DDP cells were co-transfected with Annexin A2 siRNA and p53 siRNA, and then treated with cisplatin at the indicated concentration for 48 h, cell viability was measured by MTT assay. **g** A549/DDP cells were co-transfected with Annexin A2 siRNA and p53 siRNA, and then treated with 5 μM cisplatin for 48 h, cells were stained with Annexin V-FITC and propidium iodide, and the cell apoptosis were analyzed by flow cytometry. **h** A549/DDP cells were co-transfected with Annexin A2 siRNA and p53 siRNA, and then treated with 5 μM cisplatin for 24 h, Caspase 3/7 activity were measured. *P < 0.05

It has been shown that resistance to cisplatin occurs in cells expressing low levels of p53 [24]. Therefore, we speculated whether p53 might contribute to Annexin A2-mediated cisplatin resistance. To this end, we first examined the effects of Annexin A2 on p53 expression in A549 and A549/DDP cells. As expected, siRNA-mediated Annexin A2 depletion increased the expression of both p53 mRNA and protein in a statistically significant manner in A549/DDP cells (Fig. 4a, b). Furthermore, p53-regulated apoptotic genes p21, GADD45, and BAX were also increased in Annexin A2 shRNA-transfected cells (Fig. 4b). In contrast, overexpression of Annexin A2 significantly suppressed the expression of p53 as well as p53-regulated apoptotic genes in A549 cells (Fig. 4c, d). Similar results were observed in H460 and H1650 cells (Additional file 2: Figure S5). More importantly, knockdown of p53 using p53-specific small interfering RNA (p53 siRNA) significantly decreased Annexin A2 shRNA-induced cisplatin sensitivity in A549/DDP cells (Fig. 4e, f). Flow cytometry analysis also showed that knockdown of p53 decreased the amount of apoptotic cells in Annexin A2-knockdown A549/DDP cells (Fig. 4g). Meanwhile, caspase3/7 activity in the Annexin A2-knockdown A549/DDP cells transfected with p53 siRNA was remarkably reduced than in scramble-transfected mock cells (Fig. 4h). These results clearly indicated that inhibition of p53 is critical for Annexin A2-mediated cisplatin resistance in NSCLC cells.

### Annexin A2 inhibited p53 expression and cell apoptosis through JNK/c-Jun signaling

**Fig. 5:**
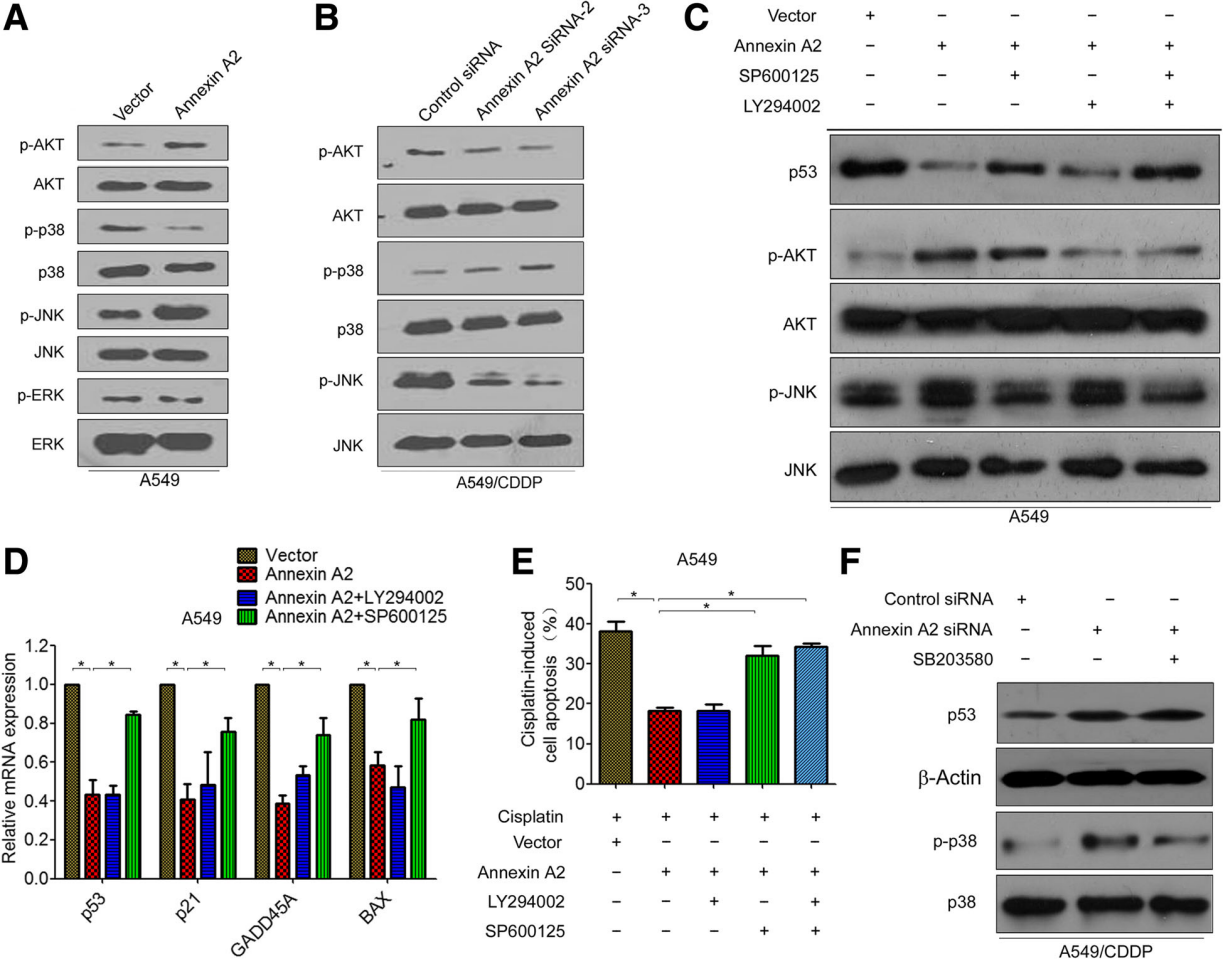
Annexin A2 inhibited p53 expression and cell apoptosis through JNK signaling. **a** A549 cells were transfected with pCMV6-Annexin A2, the expression of p-AKT, p-p38, p-JNK, p-ERK expression was analyzed by Western blot. **b** A549/DDP cells were transfected with Annexin A2 siRNA, the expression of p-AKT, p-p38, p-JNK was analyzed by Western blot. **c**-**d** A549 cells were transfected with pCMV6-Annexin A2, and then treated with JNK inhibitor SP600125 or/and AKT inhibitor LY294006, **c** the expression of p53, p-AKT, p-JNK was analyzed by Western blot; **d** the expression of p53 and p53-regulated apoptotic genes p21, GADD45, and BAX were measured by Real time RT-PCR. **e** A549 cells were transfected with pCMV6-Annexin A2, and then co-treated with SP600125 and cisplatin or co-treated with LY294006 and cisplatin, cells were stained with Annexin V-FITC and propidium iodide, and the cell apoptosis were analyzed by flow cytometry. **f** A549/DDP cells were transfected with Annexin A2 siRNA, and then treated with p38 inhibitor SB203580, the expression of p53 and p-p38 were measured by Western blot. *P < 0.05

Previously studies indicated that several relevant downstream signals, such as AKT and MAPK, might involve in tumor-promoting effects of Annexin A2 [18, 25]. Our results confirmed that overexpression of Annexin A2 significantly increased the phosphorylation levels of AKT as well as JNK, but decreased the phosphorylation levels of p38MAPK in A549 cells (Fig. 5a). However, overexpression of Annexin A2 had no effect on the phosphorylation of ERK (Fig. 5a). In contrast, we found that the levels of phosphorylation of AKT and JNK significantly decreased in Annexin A2-knockdown A549/DDP cells compared to control cells, whereas the phosphorylation of p38MAPK was increased in Annexin A2-knockdown cells (Fig. 5b).

To further investigate which pathway was required for Annexin A2-mediated inhibition of p53 and cell apoptosis, specific inhibitors targeting JNK, and AKT, and p38 MAPK pathways (SP600125, LY294002, and SB203580, respectively) were used. Western blot showed that inhibition of JNK kinase activity by SP600125 resulted in an increase in p53 protein levels in Annexin A2-overexpression A549 cells, whereas inhibition of AKT by LY294002 did not reverse the expression of p53 inhibiting by Annexin A2 (Fig. 5c). Real time RT-PCR also showed that SP600125, but not LY294002, significantly increased the mRNA expression of p53 as well as p53 targets in Annexin A2-overexpression A549 cells (Fig. 5d). More important, we found that JNK inhibitor SP600125, but not AKT inhibitor LY294002, significantly reversed cisplatin-induced cell apoptosis in Annexin A2-overexpression A549 cells (Fig. 5e). Furthermore, we found that p38MAPK inhibitor had no effect on the expression of p53 in Annexin A2-knockdown A549/DDP cells (Fig. 5f). These results suggested that activation of the JNK pathway by Annexin A2 is required for the inhibition of p53 and cell apoptosis.

**Fig. 6:**
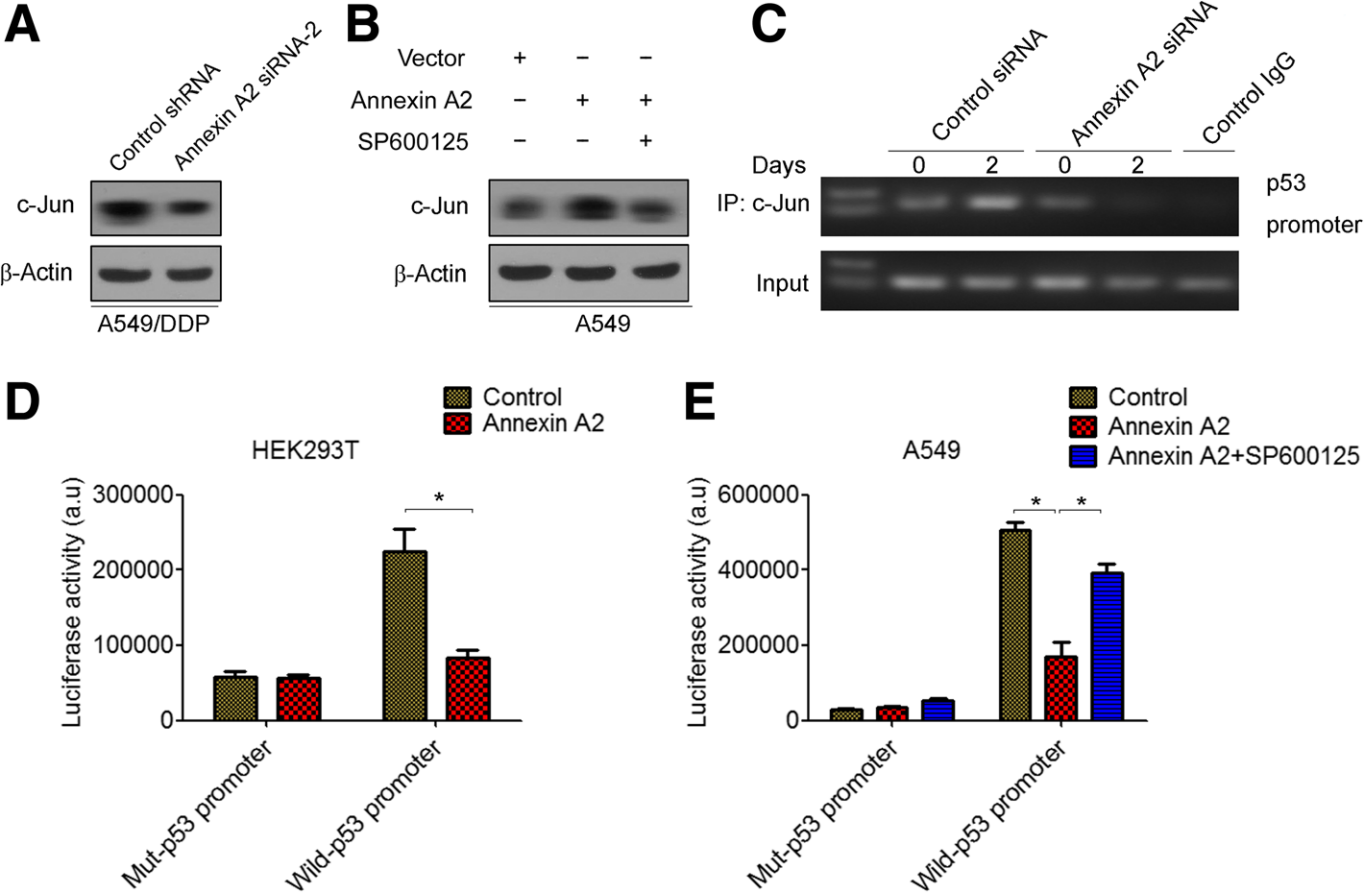
Annexin A2 inhibited p53 expression through c-Jun-mediated suppression of p53 promoter activity. **a** A549/DDP cells were transfected with Annexin A2 siRNA, c-Jun protein expression was analyzed by Western blot. **b** A549 cells were transfected with pCMV6-Annexin A2, and then treated with SP600125, c-Jun protein expression was analyzed by Western blot. **c** A549/DDP cells were transfected with Annexin A2 siRNA for 48 h, the binding capacity of c-Jun to p53 promoter were determined by chromatin immunoprecipitation. The enrichment of the p53 promoter in immunoprecipitated (IP) c-Jun and the input of c-Jun are shown. IgG was used as a negative control. One representative data set of three individual experiments is shown. **d**-**e** HEK293T cells (**d**) and A549 cells (**e**) were co-transfected with pGL3-p53-Luc, or mut-pGL3-p53 and pCMV-Annexin A2 for 48 h, cells were harvested and assayed by the Dual-Luciferase Reporter Assay System. *P < 0.05

JNK negatively regulates p53 through c-Jun stabilization [26, 27]. Thus, we tested whether Annexin A2 regulated c-Jun expression. Western blot analysis demonstrated that c-Jun was down-regulated in Annexin A2-knockdown A549/DDP cells (Fig. 6a), whereas overexpression of Annexin A2 was significantly increased the expression of c-Jun (Fig. 6b). Moreover, JNK inhibitor SP600125 decreased the expression of c-Jun inducing by Annexin A2 (Fig. 6b). p53 promoter contains a conserved AP-1-like element (termed PF-1 site) [28], and c-Jun acts as a repressor that negatively regulates p53 mRNA expression through bind and represses the p53 promoter [29, 30]. Chromatin immunoprecipitation showed that knockdown of Annexin A2 caused the loss of c-Jun binding to the p53 promoter (Fig. 6c). Furthermore, we found that Luciferase expression directed by a 700-bp fragment of the p53 promoter containing this AP-1 site is decreased in Annexin A2-overexpressing HEK293T cells (Fig. 6d). Similarity, overexpression of Annexin A2 resulted in a significant reduction of p53 promoter activity in A549 cells (Fig. 6e). In addition, we found that SP600125 significantly increased p53 promoter activity in Annexin A2 overexpressing A549 cells (Fig. 6e). Altogether, these results indicated that Annexin A2 negatively regulates p53 expression through activation of JNK/c-Jun signaling.

### Annexin A2 is overexpressed and associated with poor prognosis in NSCLCs

**Fig. 7:**
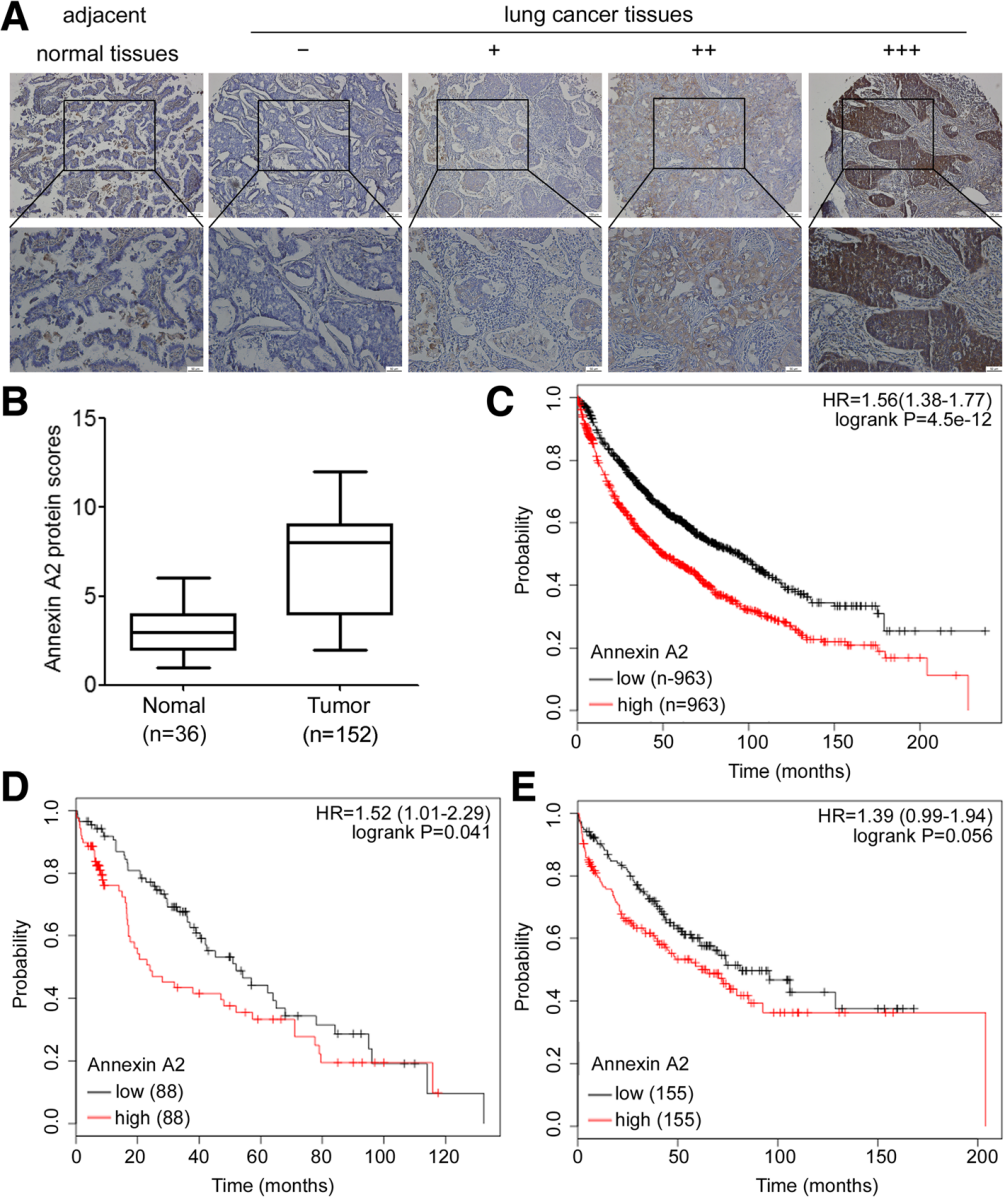
Annexin A2 is overexpressed and associated with poor prognosis in human lung cancer. **a-b** Immunohistochemistry analysis of Annexin A2 protein levels in lung cancer specimens and adjacent normal tissues. **a** Representative immunohistochemical staining examples of Annexin A2 protein expression in adjacent normal tissues and four different lung cancer tissues (Scale bar, 50 μm). The lung cancer tissue sections were quantitatively scored according to the percentage of positive cells and staining intensity as described in Materials and Methods. The percentage and intensity scores were multiplied to obtain a total score (range, 0–12), and the tumors were finally determined as negative (−), score 0; lower expression (+), score ≤ 4; moderate expression (++), score 5–8; and high expression (+++), score ≥ 9; **b** Annexin A2 expression scores in lung cancer specimens and adjacent normal tissues. *P < 0.05. **c** Kaplan–Meier OS curves (http://www.kmplot.com/lung) of 1926 lung cancer patients relative to different expression levels of Annexin A2 (probe 201590_x_at). **d** Kaplan–Meier OS curves (http://kmplot.com/analysis/) of 176 lung cancer patients administered chemotherapy relative to different expression levels of Annexin A2. **e** Kaplan–Meier OS curves (http://kmplot.com/analysis/) of 310 lung cancer patients without chemotherapy relative to different expression levels of Annexin A2

Finally, we determined Annexin A2 expression in clinical samples using immunohistochemistry analysis in 152 NSCLC tissues and 36 adjacent normal tissues and found that Annexin A2 was overexpressed in tumor samples than adjacent normal tissues (Fig. 7a, b). Next, we analyzed the relationship between Annexin A2 expression levels and clinicopathological characteristics. As shown in Table 1, no statistically significant correlations were observed between the expression of Annexin A2 and gender, or age, or lymph node metastasis at diagnosis (*p* > 0.05). However, statistically significant correlations between high levels of Annexin A2 expression were found with advanced TNM stage (*p* < 0.05). Furthermore, the association between high Annexin A2 mRNA expression and poor prognosis of NSCLCs was analyzed by using the online Kaplan–Meier survival analysis of expression data (probe 201590_x_at) from 1926 lung adenocarcinoma patients (http://www.kmplot.com/lung). The results showed that that patients with high Annexin A2 expression had a shorter overall survival time compared to patients with low Annexin A2 expression (*P* < 0.05, log-rank test) (Fig. 7c). We next focused on whether Annexin A2 expression level is correlated with the chemotherapy efficacy. We found that overall survival was shorter in patients showing a high expression of Annexin A2 than in those showing a low expression in the chemotherapy group (*P* = 0.041, log-rank test) (Fig. 7d), but not in the non-chemotherapy group (*P* = 0.056, log-rank test) (Fig. 7f).

**Table 1:** The relationship between Annexin A2 expression levels and clinical characteristics of NSCLC patients

Variables		Annexin A2		*p* Value
		Low (*n* = 45)	High (*n* = 107)	
Age	<60	21	55	0.387
	≥60	24	52	
Gender	Male	31	69	0.508
	Female	14	38	
Tumor size	<4 cm	18	37	0.212
	≥4 cm	27	70	
Histology	adenocarcinoma	25	59	0.276
	squamous cell carcinoma	20	48	
pTNM stage	I	19	12	0.012
	II-IV	26	95	
Lymph node metastasis	No	28	47	0.071
	Yes	17	60	

*p* values listed are derived from χ2 test

## Discussion

Development of drug resistance remains the major therapeutic barrier in lung cancer [31]. Therefore, identification of the molecular mechanisms underlying drug resistance is mandatory to achieve advancement in lung cancer therapy. Using a proteomic approach, we previously demonstrated that Annexin A2 might be the important factor of cisplatin resistance [20]. In this study, we showed that overexpression of Annexin A2 enhanced cisplatin resistance of A549, H460 and H1650 cells, whereas inhibition of Annexin A2 could selectively increase cisplatin sensitivity of A549/DDP cells both in vitro and in vivo, which suggested an important role of Annexin A2 in cisplatin resistance in NSCLC cells.

Aberrant Annexin A2 expression has oncogenic effects in several tumor types [7–12]. Previous studies provided evidence that in patients with lung cancer, a poor prognosis for survival is correlated with Annexin A2 expression, and this observation is consistent with the results of Annexin A2 tissue staining in lung cancer [13]. Our present data confirmed through Annexin A2 immunohistochemical staining of NSCLC tissues that Annexin A2 is overexpressed in NSCLCs and is correlation with advanced TNM stage. More important, we found that high levels of Annexin A2 is positively correlated with poor prognosis, as well as correlated with short disease-free survival for patients who received chemotherapy after surgery, which was further confirmed the specific role of Annexin A2 in chemotherapy resistance to NSCLCs.

Several mechanisms that mediate cisplatin resistance have been identified, including decreased import, pronounced activity of efflux pumps, increased detoxification, and increased efficiency of DNA repair systems [32–35]. Since DNA damage and the induction of mitochondrial apoptosis are the most critical mechanisms of cisplatin action, evasion of apoptosis might be a key feature of acquired cisplatin resistance in tumor cells [36]. Annexin A2 is involved in multiple cellular processes, including cell survival, growth, division, and differentiation. Interestingly, recent findings suggested that Annexin A2 serves as a ligand for C1q on apoptotic cells [37]. It has been showed that apoptotic stimuli induced Annexin A2 cleavage, which contributes to cell cycle inhibition and apoptosis [38], and knockdown expression of Annexin A2 made cells susceptible to chemotherapy- or radiation-induced apoptosis [38, 39]. Consistent with these results, we found that knockdown of Annexin A2 significantly increased Caspase 3/7 activity, cleaved PARP levels, as well as cisplatin-induced cell apoptosis in A549/DDP cells, which suggested that Annexin A2 enhanced cisplatin resistance of NSCLC cells by a mechanism of inhibiting cell apoptosis.

The tumor suppressor p53 is a transcription factor that regulates several genes with a broad range of functions, including DNA repair, metabolism, cell cycle arrest, apoptosis and senescence [40]. Most chemotherapeutic agents, including cisplatin, induce p53-dependent cell growth arrest and apoptosis [41]. However, when mutation or deletion of p53 renders it non-functional, drug resistance can follow [24]. Alternatively, abnormal expression of p53 regulators, such as bcl-2 and PIG3, can also lead to drug resistance [42, 43]. Based on our present results, Annexin A2 facilitates cisplatin resistance in part by inhibiting p53 expression in NSCLC cells. Consistent with this notion, Annexin A2 degradation is correlated with cellular apoptosis induced by p53-mediated pathways [44]. In response to genotoxic agents, cells depleted of Annexin A2 protected DNA from damage by enhancing phospho-histone H2Ax and p53 levels, increasing numbers of p53-binding protein 1 nuclear foci and increasing levels of nuclear 8-oxo-2′-deoxyguanine [45].

MAPK pathway activation is a common event in tumorigenesis, and plays a key role in cancer progression and invasion by regulating cell migration, proteinase induction, and apoptosis [46, 47]. In this study, we found that Annexin A2 had an effect on regulating JNK phosphorylation activation and subsequent cisplatin resistance in A549/DDP cells. We found that JNK, but not ERK1/2, was phosphorylated in A549 cells that were activated by overexpression of Annexin A2, whereas p38MAPK phosphorylation was suppressed by Annexin A2. Unfortunately, inhibition of p38MAPK is not required for Annexin A2-mediated cisplatin resistance, because p38MAPK inhibitor had no effect on the expression of p53 in Annexin A2-knockdown A549/DDP cells. The results were different from previous findings that upon loss of Annexin A2 tumor cells undergo apoptosis through activation of both JNK and p38MAPK signaling under hydrogen peroxide stimulation [48]. Moreover, similar to our results, Wang et al. found that aberrant JNK inactivation was observed in Annexin A2-knockdown cells [13]. Although JNK activation can be a positive step in the events leading a cell towards apoptosis, there are also many reports stating the opposite that activation of JNK can inhibit apoptosis and promote proliferation [49]. Specially, activation of JNK confers drug resistance of DNA-damaging agents [50–52]. Moreover, JNK is often activated in lung cancer and promotes oncogenic transformation via negatively regulating p53 through c-Jun [26, 27, 53]. Indeed, we found that activation of JNK/c-Jun signaling is essential for Annexin A2-mediated p53 suppression, as well as drug resistance. In addition, it has been shown that epidermal growth factor receptor (EGFR) activation ultimately activates JNK signaling [54, 55]. Notably, Annexin A2 interacts with EGFR at the cell surface and has an important role in cancer cell proliferation and migration by modulating EGFR functions. Blocking Annexin A2 function suppressed the EGF-induced EGFR tyrosine phosphorylation, as well as inhibited the EGFR-dependent downstream signaling pathways [56]. Therefore, EGFR may be a candidate to mediate Annexin A2-assocated JNK activation. This hypothesis needs further investigation.

## Conclusions

In summary, our results confirmed that Annexin A2 contributes to cisplatin resistance in NSCLC cells. We demonstrated that Annexin A2 suppressed p53 expression followed by p53-regulated cell apoptosis. Furthermore, we found that Annexin A2 activated JNK/c-Jun signaling, which in turn leads to an decrease in p53 transcription. Therefore, Annexin A2 could potentially be used as an important therapeutic target in drug-resistant lung cancers.

## Additional files

Additional file 1: Table S1. List of primary antibodies used in the study. (DOCX 17 kb)

Additional file 2: Figure S1. (A) Detailed 2-DE images of Annexin A2 protein spots in A549/DDP cells compared with A549 cells. (B) Mascot database search based on MALDI-TOF-MS/MS matched to Annexin A2 with highly significant scores. (C) Peptide information for Annexin A2 based on MALDI-TOF-MS/MS analysis. (D) MS/MS Fragmentation of sequence ‘GVDEVTIVNILTNR’. (E) The amino acid sequences of Annexin A2, in which matched peptide sequences are underlined. **Figure S2.** Cells were treated with cisplatin at the indicated concentration for 48 h, and cell viability was measured by MTT assay. Table indicates the IC50 values for each cell. **Figure S3.** (A) H460 and H1650 cells transfected with pCMV6-Annexin A2 were treated with cisplatin at the indicated concentration for 48 h, and cell viability was measured by MTT assay. Table indicates the IC50 values for each condition. (B) H460 and H1650 cells transfected with pCMV6-Annexin A2 were treated with cisplatin at the indicated concentration for 14 days, (Left) Colonies were fixed with acetic acid-methanol (1:4) and stained with crystal violet. (Right) The number of colonies was from three independent experiments. *P < 0.05. **Figure S4.** (A) H460 and H1650 cells were transfected with pCMV6-Annexin A2, and then treated with 10 μM cisplatin for 24 h, Caspase 3/7 activity were measured. (B) H460 and H1650 cells were transfected with pCMV6-Annexin A2, and then treated with 10 μM cisplatin for 48 h, the expression of PARP and cleaved PARP were measured by Western blot. *P < 0.05. **Figure S5.** H460 and H1650 cells were transfected with pCMV6-Annexin A2, (A) p53 protein expression was analyzed by Western blot; (B) p53 and p53-regulated apoptotic genes p21, GADD45, Bcl2, BAX, Puma, and MDM2 were measured by Real time RT-PCR. *P < 0.05 (DOCX 1720 kb)

## Abbreviations

2-DE: Two-dimensional electrophoresis
DDP: cisplatin
ERK: Extracellular signal-regulated kinase
GADD45: Growth arrest and DNA damage-inducible 45
IC50: Half maximal inhibitory concentration
JNK: c-Jun N-terminal kinase
NSCLC: Non-small cell lung cancer cell
p38MAPK: p38 mitogen-activated protein kinase
siRNA: small-interfering RNA
TNM: Tumour-Node-Metastasis
