# Supplementary material for: Annexin A2 contributes to cisplatin resistance by activation of JNK-p53 pathway in non-small cell lung cancer cells
Source: J Exp Clin Cancer Res. 2017 Sep 8;36:123. doi: 10.1186/s13046-017-0594-1 (PMC5591524; doi:10.1186/s13046-017-0594-1)
Supplement: Supplementary file 1 — List of primary antibodies used in the study. (DOCX 17 kb) [file 13046_2017_594_MOESM1_ESM.docx]

Table S1. List of primary antibodies used in the study.

| Antibody name | Source | Catalog No. | Host | Dilution |
| --- | --- | --- | --- | --- |
| Annexin A2 | Cell Signaling Technology Inc. | 8235 | Rabbit | 1:1000 |
| p53 | Cell Signaling Technology Inc. | 2524 | Mouse | 1:1000 |
| Phospho-Akt (Ser473) | Cell Signaling Technology Inc. | 4060 | Rabbit | 1:1000 |
| Akt | Cell Signaling Technology Inc. | 4691 | Rabbit | 1:1000 |
| Phospho-p44/42 MAPK (ERK1/2)  (Thr202/Tyr204) | Cell Signaling Technology Inc. | 4370 | Rabbit | 1:1000 |
| p44/42 MAPK (Erk1/2) Rabbit mAb | Cell Signaling Technology Inc. | 4695 | Rabbit | 1:1000 |
| Phospho-JNK (Thr183/Tyr185) | Cell Signaling Technology Inc. | 4668 | Rabbit | 1:1000 |
| JNK | Cell Signaling Technology Inc. | 9252 | Rabbit | 1:1000 |
| Phospho-p38MAPK (Thr180/Tyr182) | Cell Signaling Technology Inc. | 4511 | Rabbit | 1:1000 |
| p38MAPK | Cell Signaling Technology Inc. | 8690 | Rabbit | 1:1000 |
| PARP | Cell Signaling Technology Inc. | 9532 | Rabbit | 1:500 |
| β-Actin | Cell Signaling Technology Inc. | 3700 | Mouse | 1:2000 |
