# Supplementary material for: Annexin A2 contributes to cisplatin resistance by activation of JNK-p53 pathway in non-small cell lung cancer cells
Source: J Exp Clin Cancer Res. 2017 Sep 8;36:123. doi: 10.1186/s13046-017-0594-1 (PMC5591524; doi:10.1186/s13046-017-0594-1)

**Supplemental Figures S1-S5**

Figure S1. (A) Detailed 2-DE images of Annexin A2 protein spots in A549/DDP cells compared with A549 cells. (B) Mascot database search based on MALDI-TOF-MS/MS matched to Annexin A2 with highly significant scores. (C) Peptide information for Annexin A2 based on MALDI-TOF-MS/MS analysis. (D) MS/MS Fragmentation of sequence ‘GVDEVTIVNILTNR’. (E) The amino acid sequences of Annexin A2, in which matched peptide sequences are underlined.


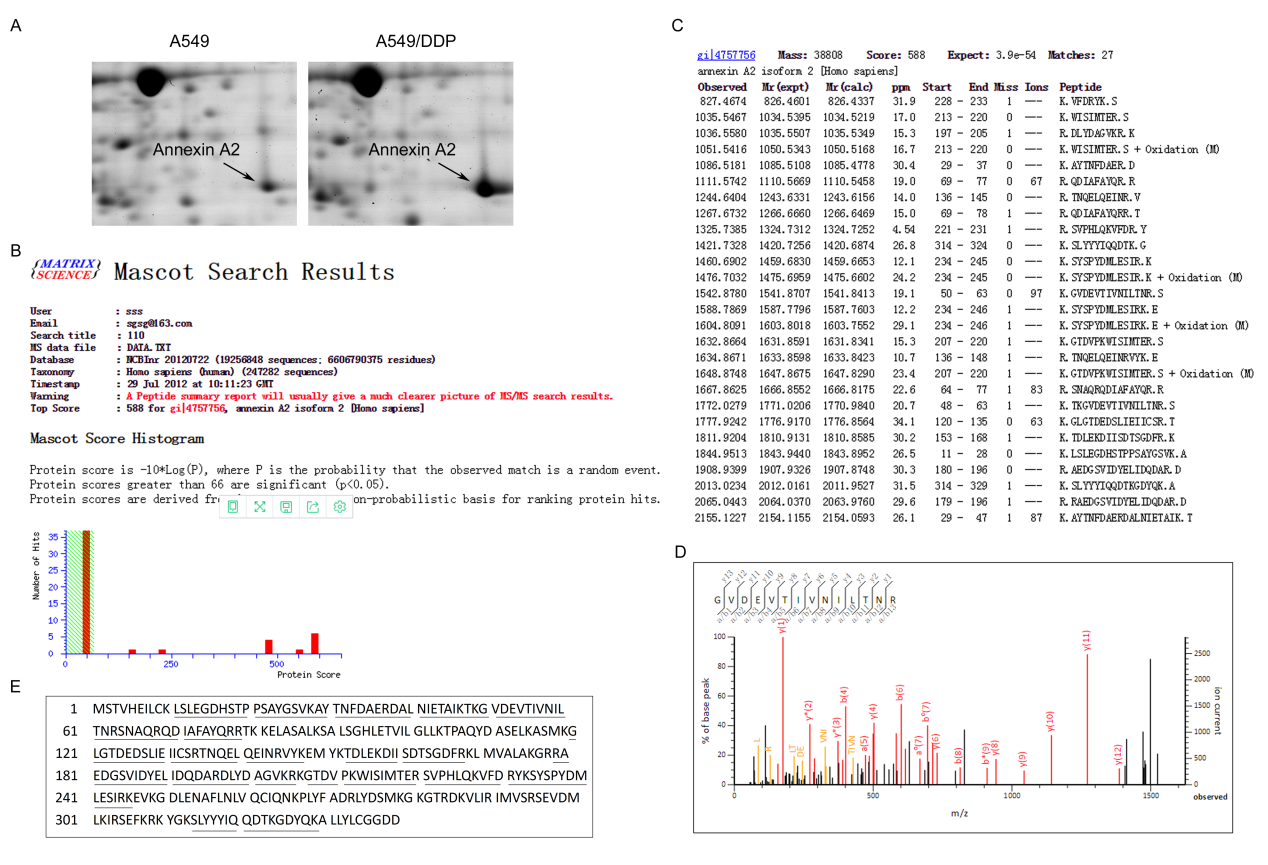


Figure S2. Cells were treated with cisplatin at the indicated concentration for 48 h, and cell viability was measured by MTT assay. Table indicates the IC50 values for each cell.


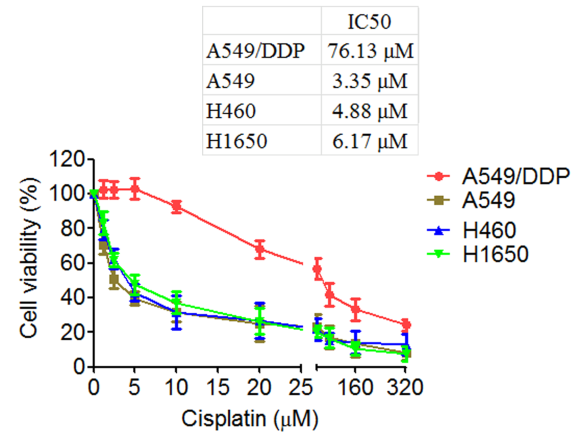


Figure S3. (A) H460 and H1650 cells transfected with pCMV6-Annexin A2 were treated with cisplatin at the indicated concentration for 48 h, and cell viability was measured by MTT assay. Table indicates the IC50 values for each condition. (B) H460 and H1650 cells transfected with pCMV6-Annexin A2 were treated with cisplatin at the indicated concentration for 14 days, (Left) Colonies were fixed with acetic acid-methanol (1:4) and stained with crystal violet. (Right) The number of colonies was from three independent experiments. *P <0.05.


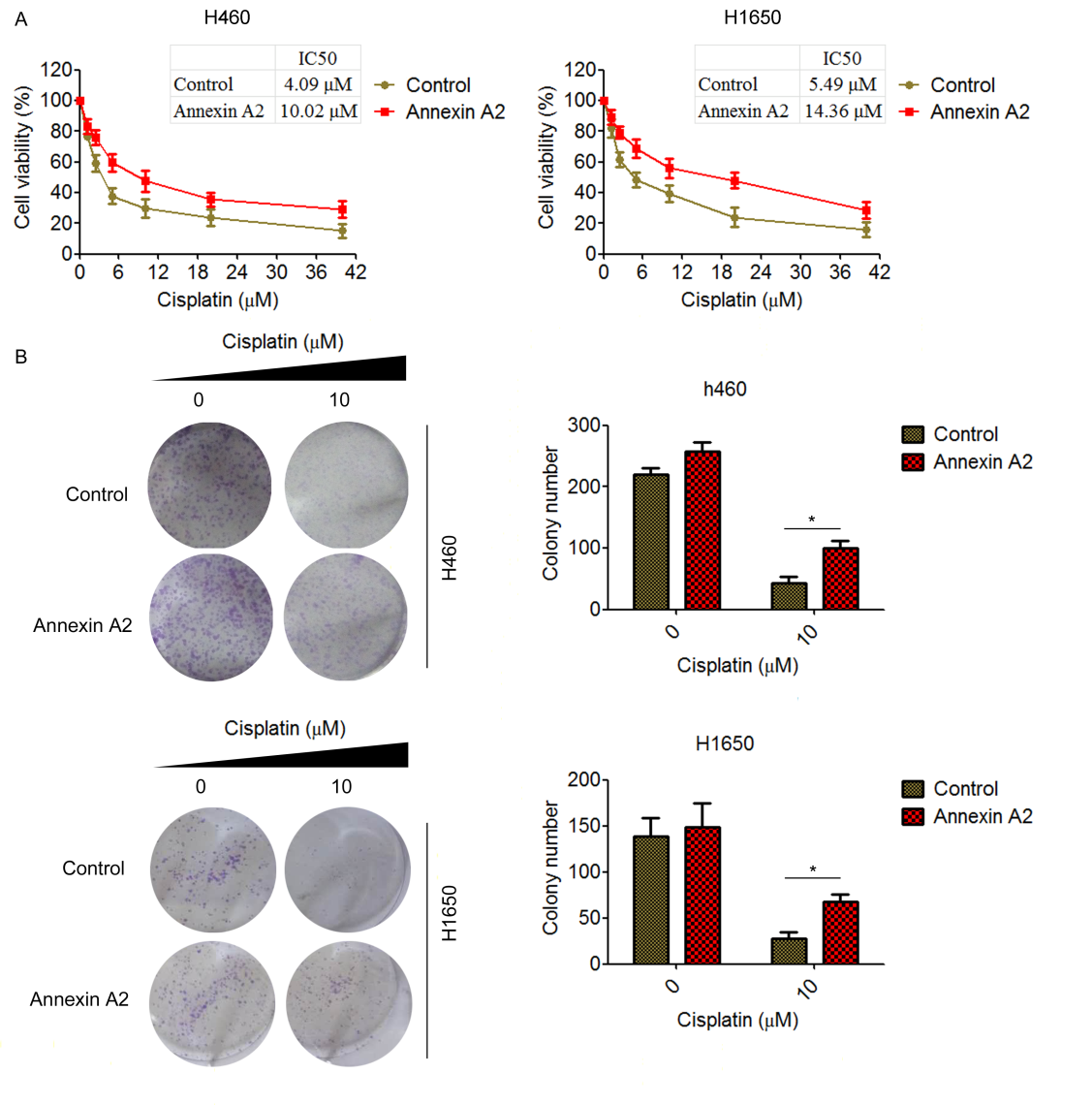


Figure S4. (A) H460 and H1650 cells were transfected with pCMV6-Annexin A2, and then treated with 10 μM cisplatin for 24 h, Caspase 3/7 activity were measured. (B) H460 and H1650 cells were transfected with pCMV6-Annexin A2, and then treated with 10 μM cisplatin for 48 h, the expression of PARP and cleaved PARP were measured by Western blot. *P <0.05.


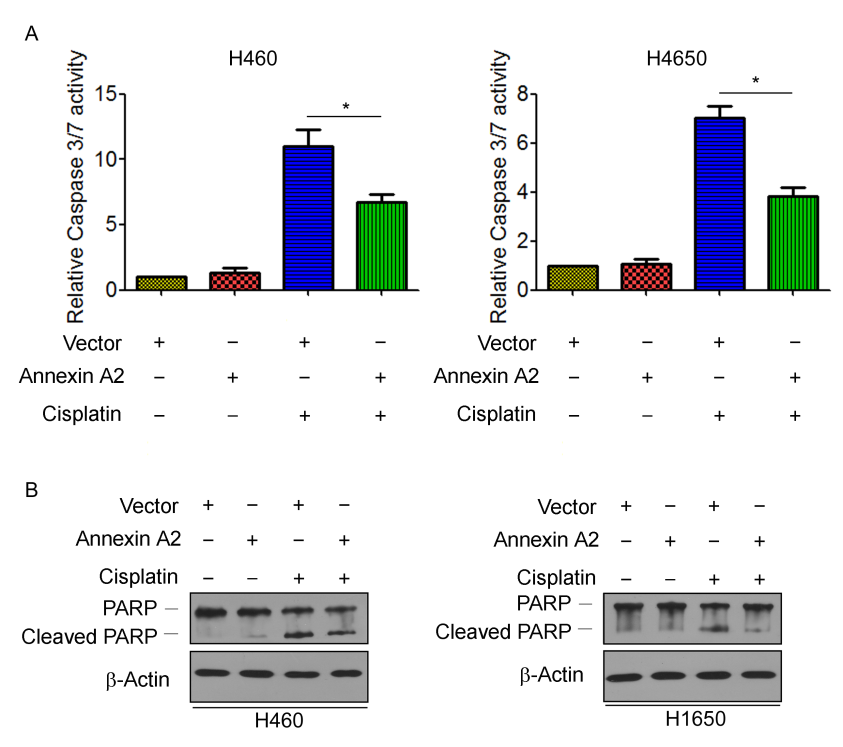


Figure S5. H460 and H1650 cells were transfected with pCMV6-Annexin A2, (A) p53 protein expression was analyzed by Western blot; (B) p53 and p53-regulated apoptotic genes p21, GADD45, Bcl2, BAX, Puma, and MDM2 were measured by Real time RT-PCR. *P <0.05.


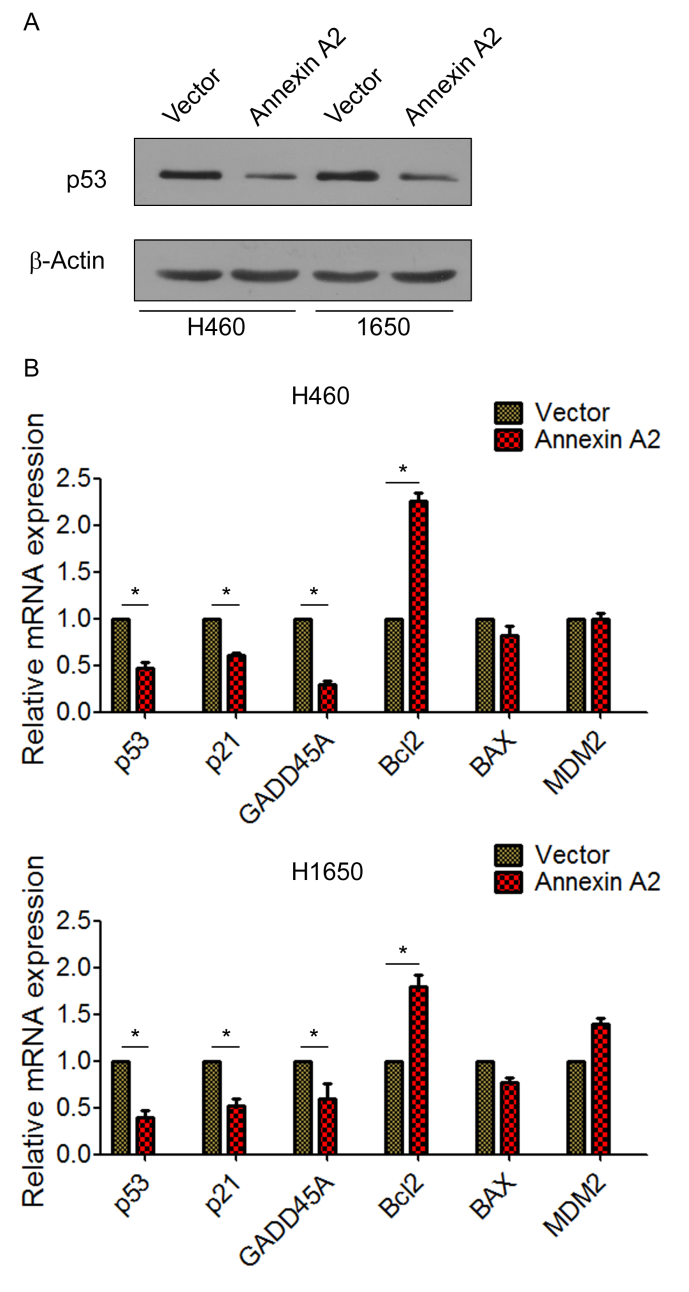

Supplement: Supplementary file 2 — (A) Detailed 2-DE images of Annexin A2 protein spots in A549/DDP cells compared with A549 cells. (B) Mascot database search based on MALDI-TOF-MS/MS matched to Annexin A2 with highly significant scores. (C) Peptide information for Annexin A2 based on MALDI-TOF-MS/MS analysis. (D) MS/MS Fragmentation of sequence ‘GVDEVTIVNILTNR’. (E) The amino acid sequences of Annexin A2, in which matched peptide sequences are underlined. Figure S2. Cells were treated with cisplatin at the indicated concentration for 48 h, and cell viability was measured by MTT assay. Table indicates the IC50 values for each cell. Figure S3. (A) H460 and H1650 cells transfected with pCMV6-Annexin A2 were treated with cisplatin at the indicated concentration for 48 h, and cell viability was measured by MTT assay. Table indicates the IC50 values for each condition. (B) H460 and H1650 cells transfected with pCMV6-Annexin A2 were treated with cisplatin at the indicated concentration for 14 days, (Left) Colonies were fixed with acetic acid-methanol (1:4) and stained with crystal violet. (Right) The number of colonies was from three independent experiments. *P < 0.05. Figure S4. (A) H460 and H1650 cells were transfected with pCMV6-Annexin A2, and then treated with 10 μM cisplatin for 24 h, Caspase 3/7 activity were measured. (B) H460 and H1650 cells were transfected with pCMV6-Annexin A2, and then treated with 10 μM cisplatin for 48 h, the expression of PARP and cleaved PARP were measured by Western blot. *P < 0.05. Figure S5. H460 and H1650 cells were transfected with pCMV6-Annexin A2, (A) p53 protein expression was analyzed by Western blot; (B) p53 and p53-regulated apoptotic genes p21, GADD45, Bcl2, BAX, Puma, and MDM2 were measured by Real time RT-PCR. *P < 0.05 (DOCX 1720 kb) [file 13046_2017_594_MOESM2_ESM.docx]
